# Genomic and transcriptomic comparison of allergen and silver nanoparticle-induced mast cell degranulation reveals novel non-immunoglobulin E mediated mechanisms

**DOI:** 10.1371/journal.pone.0193499

**Published:** 2018-03-22

**Authors:** Monica Johnson, Nasser Alsaleh, Ryan P. Mendoza, Indushekhar Persaud, Alison K. Bauer, Laura Saba, Jared M. Brown

**Affiliations:** 1 Department of Pharmaceutical Sciences, Skaggs School of Pharmacy and Pharmaceutical Sciences, University of Colorado, Aurora, CO, United States of America; 2 Department of Environmental and Occupational Health, Colorado School of Public Health, University of Colorado, Aurora, CO, United States of America; VIT University, INDIA

## Abstract

Mast cells represent a crucial cell type in host defense; however, maladaptive responses are contributing factors in the pathogenesis of allergic diseases. Previous work in our laboratory has shown that exposure to silver nanoparticles (AgNPs) results in mast cell degranulation via a non-immunoglobulin E (IgE) mechanism. In this study, we utilized a systems biology approach to identify novel genetic factors playing a role in AgNP-induced mast cell degranulation compared to the classical activation by antigen-mediated FcεRI crosslinking. Mast cell degranulation was assessed in bone marrow-derived mast cells isolated from 23 strains of mice following exposure to AgNPs or FcεRI crosslinking with dinitrophenyl (DNP). Utilizing strain-dependent mast cell degranulation, an association mapping study identified 3 chromosomal regions that were significantly associated with mast cell degranulation by AgNP and one non-overlapping region associated with DNP-mediated degranulation. Two of the AgNP-associated regions correspond to genes previously reported to be associated with allergic disorders (*Trac2* on chromosome 1 and *Traf6* on chromosome 2) and an uncharacterized gene identified on chromosome 1 (*Fam126b*). In conjunction, RNA-sequencing performed on mast cells from the high and low responder strains revealed 3754 and 34 differentially expressed genes that were unique to DNP and AgNP exposures, respectively. Select candidate genes include *Ptger4*, a gene encoding a G-protein coupled receptor in addition to a multifunctional adaptor protein, *Txnip*, that may be driving mast cell degranulation by AgNP. Taken together, we identified novel genes that have not been previously shown to play a role in nanoparticle-mediated mast cell activation. With further functional evaluation in the future, these genes may be potential therapeutic targets in the treatment of non-IgE mediated mast cell-linked disorders.

## Introduction

In the last decade, development of new nanotechnologies has contributed to the advancement of many fields. The unique properties of engineered nanomaterials (ENMs) gives them novel electrical, magnetic, mechanical, thermal, or imaging properties that are highly desirable for applications in commercial, medical, and environmental sectors [1]. Although ENMs provide many advantages, increased manufacturing and lack of safety testing raises health concerns due to increased human and environmental exposures [2, 3]. Specifically, adverse immune outcomes by ENM exposures have been reported [4].

Mast cells, a crucial cell type involved in allergic inflammation, have been shown to be activated in response to ENM exposures [5]. Specifically, exposure to a variety of physicochemically distinct ENMs including carbon-based and metal-based nanoparticles induce mast cell degranulation *in vivo* and *in vitro* [6–9]. Recently, we demonstrated a robust mast cell degranulation response following silver nanoparticle (AgNP) exposure, which was dependent on physicochemical properties such a size, shape and surface chemistry [10, 11]. AgNPs are one of the most abundantly manufactured ENM due to their antimicrobial/fungal properties and are currently utilized in more than 400 consumer products including wound dressings, food storage containers, and athletic apparel [1]. Understanding the role of AgNP exposure in immunomodulation is critical for evaluating ENM safety for consumer health, which is especially concerning for the population susceptible to mast cell mediated-diseases such as asthma, allergic dermatitis and hyperactive mast cell activation disorders [12–14].

To improve our understanding about the role of AgNPs in mast cell activation, it is crucial to determine the underlying mechanism, which is poorly defined. Conventional mast cell activation is a concerted event initiated by immunoglobulin E (IgE)-dependent cross-linking of high affinity IgE-bound receptors (i.e. FcεRI) on the surface of mast cells [15, 16]. FcεRI crosslinking by IgE and an allergen results in downstream signaling events that accumulate in the release of preformed as well as newly synthesized inflammatory mediators (i.e. histamine, proteases, leukotrienes, IL-4, IL-9, TNF-α, etc.) [13, 15]. Importantly, previous studies have shown that AgNP initiates mast cell activation via a non-IgE mediated pathway, independent of IgE sensitization [17]. Mast cell activation by non-IgE mediated mechanisms is not uncommon, as other factors have been identified to activate mast cells via alternate receptors include physical factors (pH, temperature), toxins, and endogenous signaling components [18–27]. However, the degranulation response by AgNPs has yet to be fully elucidated.

Little is known regarding the mechanism resulting in non-IgE mediated mast cell activation, however prior evidence supports a strong genetic component to allergic diseases [28, 29]. This remains particularly true in the helper T cell type 2 (T_H_2) responses (i.e. asthma, allergic inflammatory diseases) in which a number of studies have used linkage and association analysis to identify genes responsible for disease pathogenesis [30–32]. However, even less is understood about the direct genetic factors playing a role in nanoparticle-induced inflammation. Several studies utilized various strains of mice to evaluate a genetic component to this response. For example, susceptibility of quantum dot-induced lung inflammation, which resulted in neutrophil infiltration and increases in cytokines, was strain-dependent and heritable [33]. Jones et al. observed a strain-dependent effect on nanoparticle clearance, including a slower rate of clearance in strains that are prone to T cell helper type II (T_H_1; i.e. C57BL/6, B10D2) compared to T_H_2-prone (i.e. BALB/c, DBA/2) mice [34]. Overall, previous findings provide evidence that a complex set of genes regulates allergic diseases, with the potential for discovering mechanisms regulating nanoparticle-induced mast cell degranulation.

Therefore, to achieve this broader understanding of AgNP-induced mast cell activation, the current study utilized a modified hybrid mouse diversity panel consisting of 23 strains of recombinant and inbred strains of mice to determine strain-dependent susceptibility to mast cell degranulation by both AgNP and antigen-mediated FcεRI crosslinking by IgE. Using the phenotype data across multiple strains, genetic factors were elucidated using a systems biology approach. First, advanced association mapping methods were utilized to identify genetic loci associated with mast cell degranulation following AgNP exposure as well as identify novel genes mediating the classical IgE-mediated response. Lastly, transcriptomic analysis (via RNA-seq) measuring differential gene expression patterns was performed on high and low responder strains to identify novel pathways and gene targets involved in non-IgE mediated mast cell responses following AgNP exposures. To our knowledge, no gene expression data exists for murine mast cells, especially in multiple strains. Using this novel data, the current study uncovers pathways and receptors potentially playing a role in non-IgE mediated mast cell activation.

## Materials and methods

### Characterization of silver nanoparticles (AgNP)

20 nm spherical AgNPs suspended in citrate buffer were purchased from NanoComposix (San Diego, CA) at a concentration of 1 mg/ml. Primary size determined by transmission electron microscopy (TEM, Hitach H7600) & hydrodynamic diameter and zeta potential in solution were determined using ZetaSizer Nano dynamic light scattering (DLS, Malvern). All measurements were performed with 3 technical replicates at concentrations of 25 μg/ml, a dose chosen based off previously reported data [11]. It is important to note that this dose is probably higher than the average human acute exposure and is not designed to equate to human risk [35]. However, several studies have evaluated AgNP concentrations in consumer products and have demonstrated variability ranging from 1.3 μg/ml to 270,000 μg Ag /g product [35]. Dissolution of Ag^+^ was determined by ICP-MS to be 0.14 μg/ml in a solution of 25 μg/ml AgNPs in HEPES buffer for 1 hr. Importantly, we previously reported dissolution of Ag^+^ and from these particles and the ionic Ag did not contribute to mast cell degranulation (10).

### Mice

Twenty-three strains of mice were utilized in the study. An inbred panel of 18 strains of mice were chosen based on their respective genetic diversity using the ‘Hybrid Mouse Diversity Panel’ as a reference (C57BL/6J, BALB/cJ, FVB/NJ, C3H/HeJ, RIIIS/J, SWR/J, DBA/2J, 129X1/SvJ, NOD/ShiLtJ, NZW/LacJ, PL/J, CBA/J, CAST/EiJ, BALB/cByJ, LP/J, SJL/J, PWD/PhJ, I/LnJ) [36]. In addition, five recombinant inbred (RI) strains derived from parental lines C57BL/6J and DBA/2J (BXD50/RwwJ, BXD61/RwwJ, BXD73/RwwJ, BXD90/RwwJ, BXD100/RwwJ) were chosen based on the significant mast cell degranulation phenotype observed between the parental strains. Femurs from all strains were obtained from Jackson Laboratories (Bar Harbor, ME).

### Cell culture

Femoral bones isolated from 6–8 week old male mice (23 different strains as mentioned above) were used for progenitor cell isolation. Bone marrow progenitor cells (pooled from 2–3 mice) were cultured in supplemented media containing 300 ng/ml purified recombinant mouse interleukin-3 (PeproTech, Rocky Hill, NJ) for 4–6 weeks until fully differentiated into bone marrow derived mast cells (BMMCs; 37°C incubator, 5% CO_2_). The RPMI media also contained 10% FBS, 100 μg/ml streptomycin, 100 U/ml penicillin, 100 μg/ml primocin (Invivogen, San Diego, CA), 25 mM N-2-hydroxyethylpiperazine-N-2-ethane sulfonic acid (HEPES), 1.0 mM sodium pyruvate, nonessential amino acids (BioSource International, Camarillo, CA), and 0.00035% 2-mercapto-ethanol. All animal procedures were approved and conducted in accordance with the National Institutes of Health guidelines and approved by the University of Colorado Denver Institutional Animal Care and Use Committee. Cells were maintained in non-adherent flasks until treatment. To confirm mast cell responses in a human model, we utilized LUVA cells, a human mast cell line, which was obtained from Kerafast (Boston, MA). Cells were cultured at 37°C and 5% CO_2_ in StemPro^®^-34 SFM Complete Medium (Thermo Fisher Scientific Waltham, MA) containing 200 mM L-glutamine, 100 U/ml penicillin, 100 μg/ml streptomycin, 100 μg/ ml Primocin^TM^. Cells were plated at 75,000 cells per well in HEPES, exposed to AgNPs at 25 or 50 μg/ml for 1 hour, and then cell degranulation was assessed as described below.

### Flow cytometric analysis

To assess surface receptor expression, BMMCs were incubated with the following antibodies (Thermo Fisher Scientific) for 30 min at room temperature: PE-conjugated anti-FcεRI (MAR-1) and FITC-conjugated anti-CD117 (c-Kit). Flow cytometric analysis was performed on 1 x 10^5^ cells performed in triplicate from 3 individual batches of BMMCs using an Accuri C6 Flow Cytometer (BD Biosciences, San Jose, CA) with FCS Express 4 software (De Novo software).

### Mast cell degranulation assay

BMMCs cultured for 4–6 weeks were plated at confluency at 5 x 10^4^ cells per well in non-adherent plates in biological buffer (HEPES, pH 7.4). β-hexosaminidase (β-hex) enzyme release was analyzed in BMMC and LUVA human mast cells following exposure of dinitrophenyl (DNP, 100 ng/ml, 30 min), AgNP (25 μg/ml, 1 h) with or without pretreatment (30 min) of the selective competitive antagonist of the EP_4_ receptor (10 μM, GW627368X, Cayman Chemicals), prostaglandin E (PGE_2_, 10 μM, Sigma) or non-selective inhibitor of cyclooxygenase (COX)- 1 and -2 (indomethacin, 10 μM, Sigma) as previously described [10, 37]. For DNP-mediated degranulation, BMMCs were sensitized overnight with IgE anti-DNP (100 ng/ml, Sigma- Aldrich, St. Louis, MO), followed by treatment with DNP-HSA for 30 min. After 30 min incubation of DNP-HSA or 1h incubation of AgNPs (25 μg/ml), p-nitrophenyl-N-acetyl-ß-D-glucopyranoside (PNAG; Sigma–Aldrich, St. Louis, MO), a chromogenic substrate of ß-hexosaminidase, was added to cell supernatants and lysates and incubated for 90 min at 37°C. The reaction was stopped with glycine (0.4M) and optical density was read at 405 nm using a Synergy^TM^ HT Multi-Mode Microplate Reader (BioTek Instruments Inc, Winooski, VT). ß-hex release was calculated as percent total cell release after subtracting background release from untreated cells and normalized to non-treated control cells. Basal levels of ß-hex were similar across all strains examined. All experiments were performed in triplicate from 3 individual batches of mature mast cells grown in IL-3 supplemented media.

### Statistical analyses

Graphs for mast cell maturation and degranulation were designed and analyzed using GraphPad Prism 7 software (San Diego, CA). Mast cell degranulation measurements are represented as mean technical replicates of all biological replicates (n = 2–3) ± standard error of the mean (SEM). Statistical comparisons were performed by Student’s *t*-test for two groups and analysis of variance (ANOVA) for more than two groups, with differences between groups assessed using a Bonferroni *post hoc* test.

The heritability of mast cell degranulation by AgNP and DNP was estimated from the variance components of the linear mixed model that accounts for population structure. The pylmmGWAS multiPhHeri.py program distributed with the MultiTrans software was used to estimate these variance components [38].

### Inbred and RI genotyping

For the association mapping, single nucleotide polymorphism (SNP) information on the 23 strains of mice was downloaded from the Mouse Phenome Database (Jackson laboratories, http://phenome.jax.org, [39, 40]. Of the 415,431 fully informative SNPs retrieved after removing heterozygous calls or missing genotyping data, 198,345 informative SNPs with a minor allele frequency greater than 10% and positions within the NCBI-build-38 version of the mouse genome were used for association mapping.

### Association mapping

Genome-wide association mapping was performed using phenotype data from mast cell degranulation assays following either AgNP or DNP treatment as described above. Association mapping was executed using the linear efficient mixed-model association (emma) package (version 1.1.2) in R statistical software (version 3.3.0), taking into account population structure among strains and variance between biological replicates [41]. One strain, BALB/cByJ was not included in the mapping study due to its high degree of genetic similarity to BALB/cJ. The genome-wide significance threshold was calculated using a Bonferroni adjustment for the number of unique strain distribution patterns within the SNP data. Upper and lower bounds of an associated region were defined as the location of the most proximal and most distal genotyped SNP nominally association with the genotype (unadjusted *p*-value<0.01) within 10 Mb of a SNP that reached genome-wide statistical significance (Bonferroni adjusted *p*-value<0.05). *P*-values from association mapping were–log_10_ transformed for graphic visualization.

The UCSC Genome Browser (https://genome.ucsc.edu) was used to identify genes residing in the associated regions [42]. The NHGRI-EBI GWAS catalog (https://www.ebi.ac.uk/gwas) was utilized to find any candidate genes that had been previously identified in human GWAS for allergic immune response traits (i.e. asthma, allergies, allergic dermatitis, general immunological responses) [43].

### RNA-sequencing

High and low responder strains were selected based on their differences in mast cell degranulation response following AgNP (i.e. C57BL/6, LP/J) or DNP (i.e. RIIIS/J, C57BL/6J) treatment. For each strain, two biological replicates of BMMCs were produced. Within a biological replicate, cells were separated into 2 groups (3 groups for C57BL/6J), one group remained untreated and the other group was treated with either AgNP (25 μg/ml, 1h) or DNP (100 ng/ml, 30 min) as described above. Following treatment, total RNA from BMMCs was isolated using TRIzol reagent (Invitrogen, Carlsbad, CA, USA) and purification using a Direct-zol RNA miniprep kit (Zymo Research) following the manufacturers protocol. RNA quality and quantity was estimated by QC (Agilent 2100 Bioanalyzer, Santa Clara, CA). The detailed mRNA library preparation was performed following the manufacturers protocol using TruSeq Stranded mRNA Sample Preparation kit (Illumina, San Diego, California). The library products were sequenced on the Illumina HiSeqTM4000 (Illumina; 50 cycles, single-read sequencing, 7 samples per lane). Raw sequencing reads were trimmed for adapters and for quality using the default settings of *trim_galore* (http://www.bioinformatics.babraham.ac.uk/projects/trim_galore/). Trimmed reads were aligned to the Ensembl mouse transcriptome (version GRCm38.84) using the RSEM (RNA-Seq by Expectation-Maximization) package (v1.2.31) [44]. Gene-level expected counts were used for differential expression analysis with the DESeq2 R package (version 1.12.2). Prior to differential expression analysis, genes were removed if their average expected read count across the 14 samples was less than 5. Two separate models were used to examine the effects of AgNP and DNP. For each treatment type, we examined the effect of the specific treatment compared to control animals by initially producing a single *p*-value that simultaneously tested for either common treatment effect across strains or a strain-specific treatment effect within each gene in a model that also included an effect for the animal from which the mast cells were derived. We also specifically tested for difference in treatment effects between inbred mouse strains. The differences in expression levels were estimated from regularized log transformed values of the original count data (*rlog* function in the DESeq2 package). A false discovery rate (FDR; [45]) was used to adjust for multiple comparisons across genes. Statistical overrepresentation tests of gene ontology (GO) categories enriched in the differentially expressed gene sets were analyzed using Panther Classification System (http://.pantherdb.org/, [46]). The binomial test compares an input (test) gene list to a reference gene list and determines whether there is statistical overrepresentation or underrepresentation of categories in the input gene set. ‘Expected’ values are the number of genes that would be expected in a gene list given the number of genes mapped to a specific GO category and ‘actual’ values are the input genes. Significance was set at *p*-*value* cut off of 0.05 with a fold enrichment cutoff of 5.

### Quantitative real-time polymerase chain reaction (qPCR)

Following 1 h treatment of AgNP (25 μg/ml), total RNA from BMMCs was collected as described above. RNA was reverse-transcribed using the Quantitect reverse transcription kit (Bio-Rad, Hercules, CA). Quantitative real-time polymerase chain reaction (qPCR) was performed using a Quantitech SYBR Green PCR kit (Bio-Rad) and the StepOne Plus real-time PCR system (Applied Biosciences) to obtain cycle threshold (C_t_) values for target and internal reference cDNA levels. Gene specific primers for *Ptger4*, *Txnip*, *Trib2*, *Fam43a*, and *Gadd45g* were obtained from Invitrogen. Gene expression levels were calculated relative to *Gapdh* (glyceraldehyde 2-phosphate dehydrogenease) as an internal endogenous control according to the ΔCT method where ΔC_t_ is defined as C_t target_- C_t internal reference_. Values are represented as the mean ± SEM from 3 individual batches of mature mast cells grown in IL-3 supplemented media.

### Western blotting

Protein expression analysis by western blotting was performed similar to previously described methods [17]. Briefly, BMMCs isolated from high responder (C57BL/6) and low responder (LP/J) strains of mice were exposed to AgNP at 25 μg/ml for 1 hour. Following isolation of cell supernatant by lysis, protein concentration was determined using Pierce BCA protein assay kit (Thermo Scientific #23225). 20 μg of total protein was loaded and separated using a 12% Tris-Glycine gel and transferred onto nitrocellulose membrane. Primary goat anti-mouse antibodies for β-actin and TXNIP (Cell Signaling Technologies), were incubated overnight, followed by incubation of horseradish-peroxidase conjugated secondary antibodies. Membranes were developed using Pierce Chemiluminescent Substrate (Thermo/Fisher Scientific, Waltham, MA). Relative densitometry was evaluated using Image J software (NIH, Bethesda, MD, USA). Immunoblots are representative images from three independent experiments.

## Results

### Characterization of silver nanoparticles (AgNP)

Primary size of silver nanoparticles (AgNP) at 25 μg/ml as determined by transmission electron microscopy was 20 ± 4.2 nm. In solution, the hydrodynamic diameter measured by dynamic light scattering measured 16 ± 1.1 nm. The zeta potential was -38 mV, indicating high stability in solution.

### Genetic effects of mast cell degranulation caused by silver nanoparticles (AgNP) or FcεRI crosslinking by dinitrophenyl (DNP)

**Fig 1:**
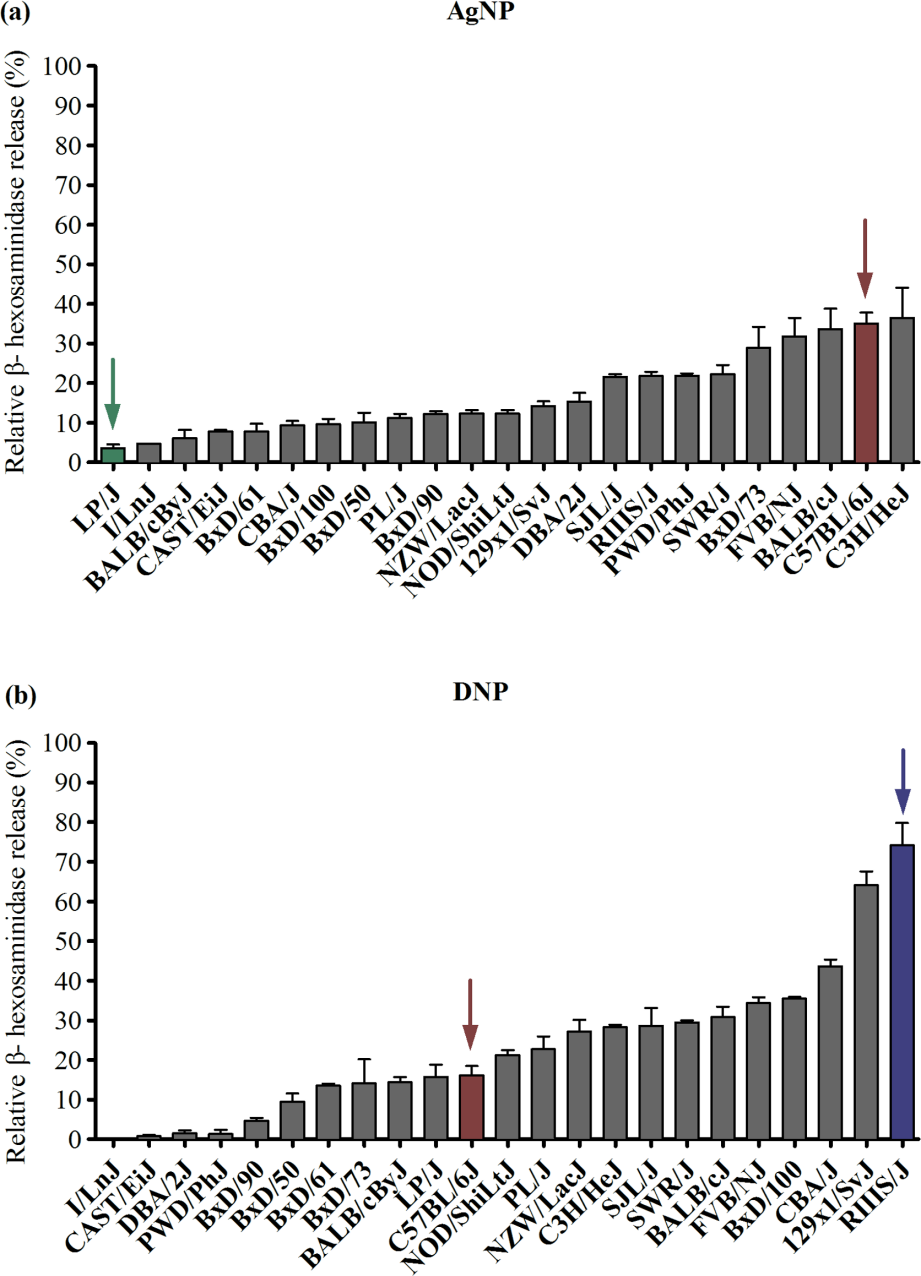
Mast cell degranulation by AgNP and DNP across 23 strains of mice. Bone marrow-derived mast cells (BMMCs) were isolated from 23 inbred and recombinant inbred (BxD) strains of mice. Degranulation was assessed by measuring ß-hexosaminidase release following (A) AgNP treatment at 25 μg/ml for 1 h in un-sensitized BMMCs, or (B) DNP treatment at 100 ng/ml for 0.5 h after BMMC overnight sensitization with IgE anti-DNP. Arrows represent low and high responder strains for each treatment used in RNA-seq (AgNP: green-LP/J, red-C57BL/6J; DNP: red-C57BL/6J, blue-RIIIS/J). Values are expressed as mean ± SEM (n = 3/strain) normalized to strain-specific non-treated control values.

To characterize the genetic contribution to mast cell responses, primary bone marrow derived mast cells (BMMCs) were isolated from 23 strains of mice and evaluated for degranulation by measuring the release of ß -hexosaminidase following FcεRI crosslinking by DNP or AgNP (25 μg/ml) treatment (Fig 1). Prior to degranulation experiments, BMMC maturation was confirmed by expression of CD117 (c-Kit) and the high affinity immunoglobulin E (IgE) receptor (FcεRI)). All strains except I/LnJ, PWD/PhJ, DBA/2J, and CAST/EiJ fully expressed both receptors at 5 weeks following culture in IL-3 (S1 Fig). Strain differences in mast cell degranulation following DNP stimulation revealed a strong genetic influence (heritability = 96%; Fig 1B). For example, the highest responder strain, RIIIS/J (74%) had a 3.6-fold increase in mast cell degranulation compared to C57BL/6J (16%), which is a commonly used strain in mast cell studies. Four non-responsive strains: I/LnJ, PWD/PhJ, DBA/2J and CAST/EiJ had nominal responses below 2% degranulation as expected due to low FcεRI and c-Kit receptor expression.

Interestingly, mast cell degranulation by AgNP also exhibited a strong genetic influence (heritability = 83%) but followed a strikingly different strain distribution pattern compared to DNP (Fig 1B). C3H/HeJ (36%) and C57BL/6J (35%) were the highest responder strains with an 8-fold increase in response compared to the lowest responder strain, LP/J (4%). The four strains previously identified with low surface receptor expression and subsequently non-responsive to DNP-induced mast cell degranulation (I/LnJ, PWD/PhJ, DBA/2J, CAST/EiJ), displayed very different responses following AgNP treatment. Specifically, AgNP treatment caused significant degranulation in the two strains PWD/PhJ (22%) and DBA/2J (15%). These results further demonstrate that AgNPs are not activating mast cells via an FcεRI mechanism but likely a novel receptor or cell membrane-driven mechanism. Lastly, we confirmed the mast degranulation response using LUVA human mast cells. As shown in S2 Fig, a dose dependent increase in mast cell degranulation was observed following AgNP treatment, consistent with murine mast cell responses.

**Fig 2:**
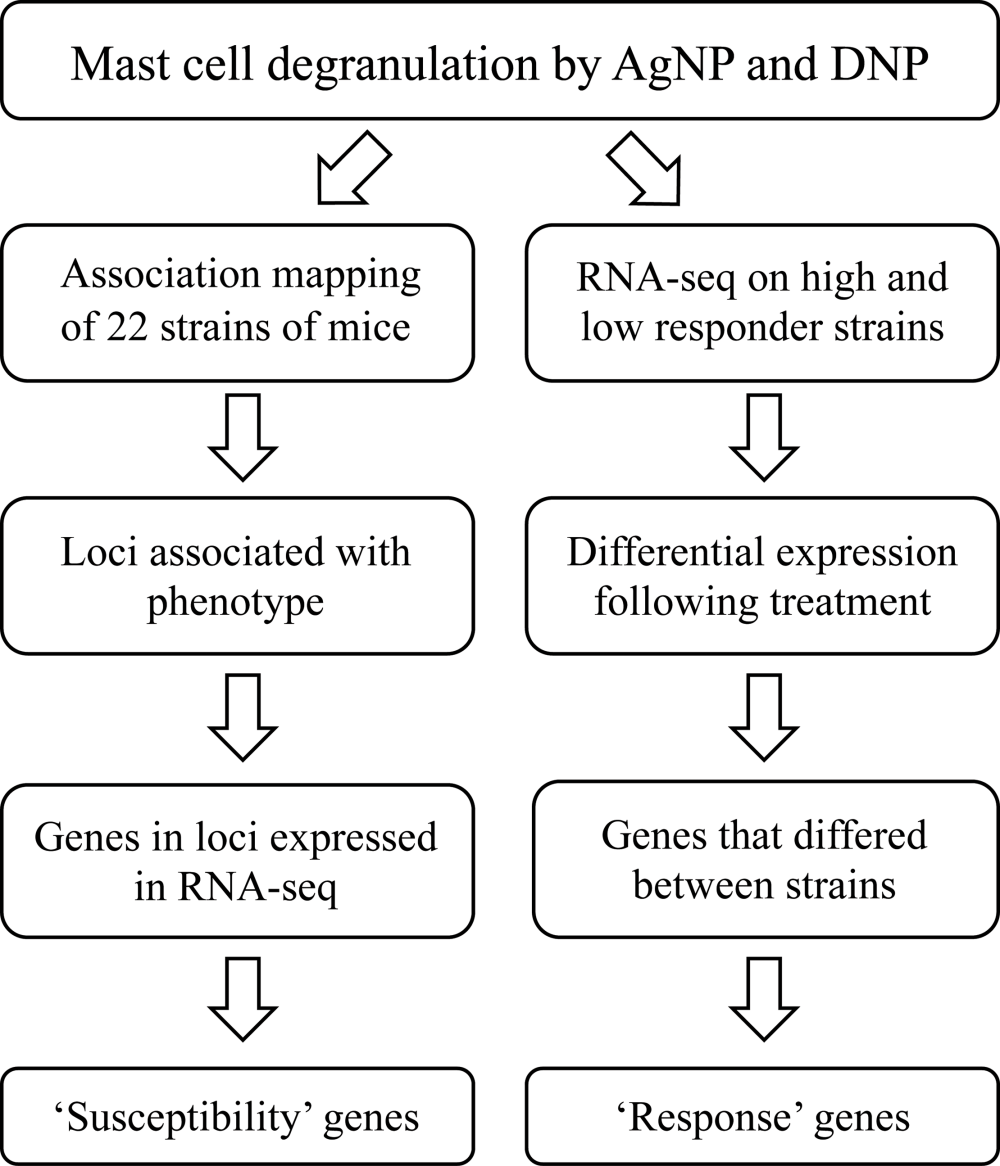
Systems biology workflow. Association mapping study utilizing 22 strains of mice were performed in conjunction with RNA-sequencing on high and low responder strains. ‘Susceptibility’ and ‘response’ genes were reported.

Based on the unique strain-dependent differences in mast cell degranulation following FcεRI crosslinking by DNP as compared to non-IgE-mediated AgNP exposure, we utilized a systems biology approach (outlined in Fig 2) to:

1. Map genetic regions/loci and report “susceptibility” genes that are associated with differential mast cell degranulation via a non-IgE mediated (AgNP) mechanism
2. Identify “response” genes that are differentially expressed following treatment to better understand the unique transcriptomic effect of AgNP compared to DNP
3. Elucidate novel mechanisms of mast cell activation, which may be important in identifying potential targets involved in non-IgE mediated mast cell activation commonly observed in idiopathic disorders

### Association mapping identifies unique susceptibility genes involved in mast cell degranulation

**Fig 3:**
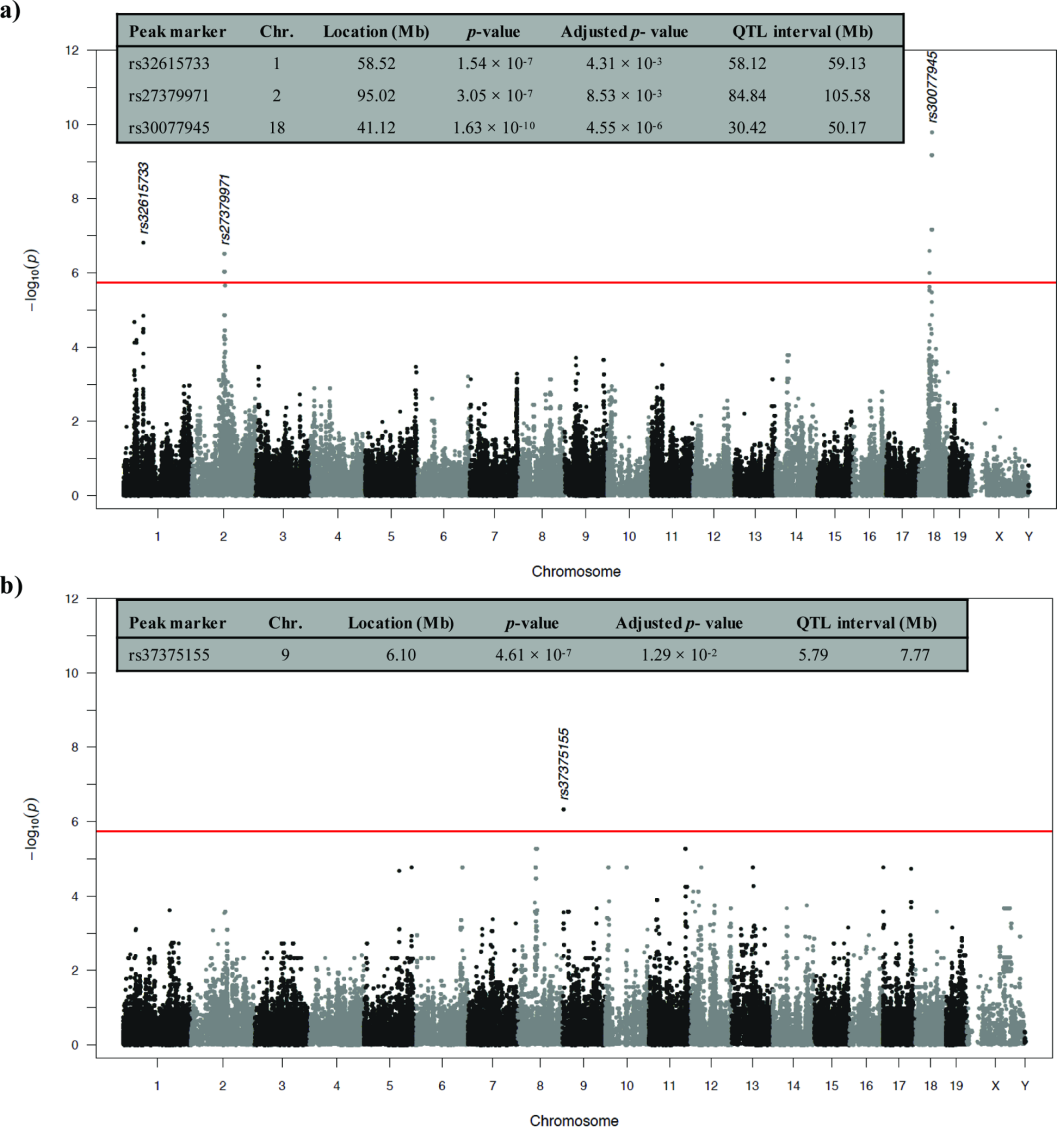
Association mapping of AgNP or DNP treatment with mast cell degranulation. Manhattan plot of chromosomal loci associated with (A) AgNP or (B) DNP treatment across 22 strains of mice. The figure inserts include further information on the peak marker, chromosome, *p*-values, and associated regions. Loci with *p*-values ≤ 7×10^−5^ (red line) and adjusted *p*-values ≤ 0.05 were considered significant.

An association mapping study was performed on 22 strains of mice surveyed for mast cell degranulation. Using an efficient mixed-model approach (emma) on 198,278 informative SNPs, we identified chromosomal loci associated with quantitative differences in mast cell degranulation by AgNP and DNP (Fig 3). The mapping study identified three loci associated with AgNP-induced mast cell degranulation, which were mapped on chromosomes 1, 2, and 18 (Bonferroni corrected *p*-value: 4.3 × 10^−3^, 8.5 × 10^−3^, 4.6 × 10^−6^, respectively) (Fig 3A). One locus was associated with DNP-mediated mast cell degranulation on chromosome 9 (Bonferroni corrected *p*-value: 1.3 × 10^−2^) (Fig 3B). All associated loci exceeded a significance threshold of Bonferroni-corrected *p-*value <0.05. Genes residing in any of the 4 associated regions between both treatments were narrowed by only including genes that were expressed in the transcriptomic data (Table 1). The ≤20 closest genes to the SNP peak marker for each locus are outlined in Table 1. Utilizing the NHGRI-EBI catalog of previously reported GWAS, candidate genes were further narrowed to include QTLs and genetic variants previously associated with allergic immune response traits. Genes of interest are trafficking protein, kinesin binding 2 (*Trak2)* on chromosome 1 and TNF receptor–associated factor 6 (*Traf6)* on chromosome 2 because they were discovered together in several previously reported GWAS studies associated with atopic dermatitis, psoriasis, and rheumatoid arthritis [47–49].

**Table 1:** Chromosomal loci significantly associated with mast cell degranulation by AgNP or DNP and genes residing in each locus.

Treatment	Chr.	QTL interval (Mb)	Peak marker	Location (Mb)	Adjusted *p*-value	# of genes in region ^a^	Genes closest to peak marker^b^
**AgNP**	1	58.12–59.13	rs32615733	58.52	4.31 × 10^−3^	13	*NDUFB3*^*59*^, *NIF3L1*^*59*^, *FAM126B*^*59*^, *BZW1*^*59*^, *CASP8*^59^, *CFLAR*^*59*^, *CLK1*^*59*^, *PPIL3*^*59*^, *TRAK2*^*47-49*^, *STRADB*, *MPP4*, *ORC2*
	2	84.84–106.38	rs27379157	96.49	8.53 × 10^−3^	79	*PRDM11*, *CREB3L1*, *TRAF6*^*47-49*^, *LDLRAD3*^*54*^, *CD82*^*50*^, *API5*, *TTC17*, *HSD17B12*, *ALKBH3*, *ACCS*, *EXT2*, *TRP53I11*, *MAPK8IP1*, *SLC35C1*, *CHST1*, *PEX16*, *PHF21A*, *CRY2*
	18	30.42–50.19	rs30077945	41.12	4.55 × 10^−6^	100	*GNPDA1*, *RNF14*, *NDFIP1*^*50*^, *LARS*, *TCERG*, *ARHGAP26*, *PRELID2*, *SH3RF2*, *YIPF5*, *RBM27*, *EIF3J2*, *NR3C1*, *HDAC3*, *ARAP3*, *YTHDC2*, *PCDH1*
**DNP**	9	5.79–7.77	rs37375155	6.1	1.29 × 10^−2^	9	*DYNC2H1*, *MMP8*, *MMP7*, *DCUN1D5*^*52*^, *MMP12*, *MMP13*, *TMEM123*, *MMP27*, *GM10709*

^a^ Indicates # of genes residing in the locus that were also identified in RNA-sequencing analysis.

^b^ Protein-coding genes (<20) closest to each peak marker residing in the locus. Proposed candidate genes identified in other GWAS studies are bold and genes overlapping with peak marker are underlined.

Chr., chromosome; AgNP, silver nanoparticles; DNP, dinitrophenyl.

### Transcriptomic analysis of high and low responder strains distinguishes novel response genes in AgNP-induced mast cell degranulation

RNA sequencing (RNA-seq) was performed on BMMCs grown from the high and low responder strains following DNP (RIIIS/J, C57BL/6J) or AgNP (C57BL/6J, LP/J) mediated degranulation. The filtered data set comprised approximately 750 million—50 base pair reads, almost evenly distributed between analyzed samples. Of the 16,504 genes from *Mus musculus* reference transcriptome that were expressed in mast cells, 3805 and 85 protein-coding genes were differentially expressed (DE) following DNP or AgNP treatment, respectively (FDR<0.05). Of the total 3890 DE genes, 2702 (DNP) and 21 (AgNP) genes displayed DE patterns that differed between the high and low responder strains (strain-dependent treatment effect; FDR<0.1). A complete list of all DE genes following AgNP and DNP treatment are outlined in the supplemental material (S1 Table).

**Fig 4:**
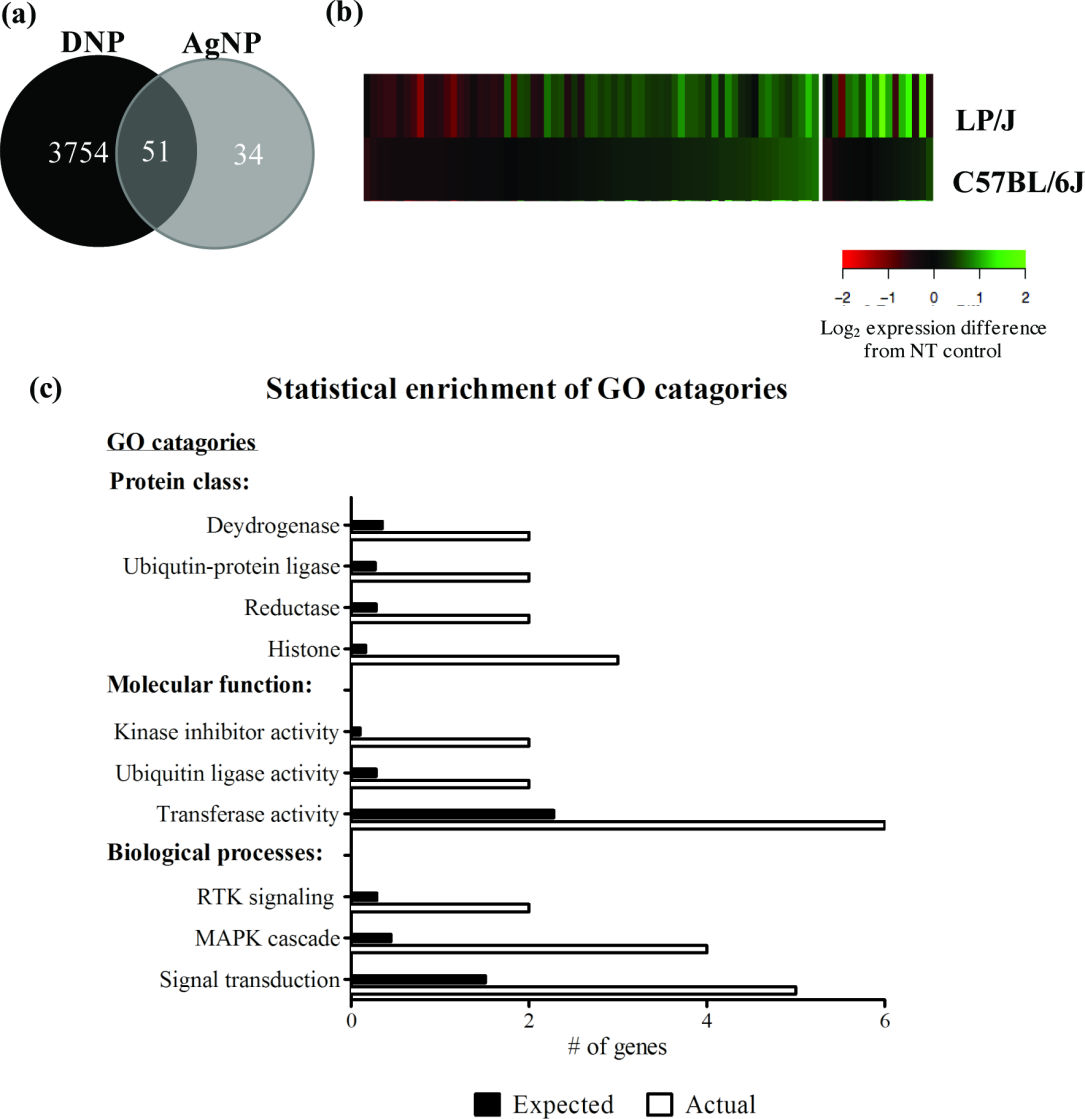
Quantitative comparison of differentially expressed transcripts and the biological responses that are significantly enriched in mast cells by AgNPs. (A) Venn diagram showing the number of differentially expressed (DE) genes produced in response to DNP treatment (black) and AgNP treatment (grey) with a combined total of 3890 genes. (B) Heat map of the relative expression of 85 DE genes (FDR≤0.05) following AgNP exposure in the low responder (LP/J) and high responder strain (C57BL/6J). Expression patterns for individual genes are in rows. The color represents the direction of difference in expression with red indicating increased expression and green indicated decreased expression after treatment; The intensity of the color represents the magnitude of difference in expression (log_2_ scale) between AgNP treated cells and non-treated cells. (C) Gene ontology (GO) categories overrepresented in the 34 AgNP-responsive gene set were assessed using Pantherdb.org. GO categories are ranked by number of genes, listing the number of AgNP-responsive genes in each category (white bar) verses the number of genes expected in each category (black bar). Significance was set at *p*≤0.05 with a fold enrichment cutoff of ≥5. RTK, receptor tyrosine kinase; MAPK, mitogen-activated protein kinase.

Quantitative comparison of the transcriptomic effects due to AgNP and DNP are reported in Fig 4. A large majority of the total DE genes following both treatments were unique to DNP treatment (3754 out of 3890) (Fig 4A). Interestingly, 34 genes were DE following AgNP treatment, which were further evaluated as novel genetic targets involved in non-IgE-mediated mast cell activation. We examined global expression patterns for the DE genes following AgNP treatment using cluster analysis (Fig 4B). Significant variability in expression of individual genes within treatment groups was apparent. The heat map clearly outlines the abundance of DE genes observed in the low responder stain (LP/J) with marginal expression changes observed in the high responder strain (C57BL/6J), potentially indicating that many DE genes were protective in response to AgNP treatment (Fig 4B). Taken together, these initial analyses revealed that AgNP treatment has specific and different transcriptomic effects compared to DNP treatment, and the AgNP effects were further differentiated between the low (LP/J) and high responder (C57BL/6J) strains.

We focused further studies on the candidate gene list comprised of 34 AgNP-responsive genes (Table 2). Of specific interest are the 11 DE genes in which AgNP-induced contrasting responses in the high versus the low responder strains (Table 2, bold). We tested for gene ontology (GO) categories overrepresented in the gene set to identify unique biological responses of mast cells to AgNP (Fig 4C). Treatment of BMMCs with AgNP had a strong, positive effect on a number of fundamental and interconnected biological processes involved in stress response including histone modifications and signal transduction pathways. Specifically, several histone components (*Hist1h1d*, *H3f3b*, *Hist3h2a*), signal transduction components including the MAPK signaling cascade (*Trib2*, *Ptger4*, *Fam43a*), and stress response genes involved in kinase inhibitor activity and ubiquitin protein ligase activity (*Txnip*, *Arrdc3*, *Arrdc4*, *Gadd45g*, *Neurl3*) were significantly enriched in the AgNP-responsive genes. These results largely confirmed that AgNP-induced mast cell degranulation is a complex process that is likely mediated through a novel, receptor-driven mechanism leading to activation of cellular stress response pathways ultimately culminating in degranulation.

**Table 2:** Candidate genes identified by RNA-seq that are differentially expressed following AgNP treatment.

Gene name^a^	Gene description	FDR^b^	Fold change^c^	
			C57BL/6J	LP/J
***Txnip***	**Thioredoxin interacting protein**	**3.73 × 10**^**−88**^	**1.08**	**4.15**
*H3f3b*	H3 histone, family 3B	6.19 × 10^−17^	1.3	1.76
***Rsad1***	**Radical S-adenosyl methionine domain containing 1**	**1.18 × 10**^**−15**^	**1**	**2.35**
***Arrdc3***	**Arrestin domain containing 3**	**2.63 × 10**^**−12**^	**1.08**	**1.99**
***Gm4737***	**Predicted gene 4737**	**3.04 × 10**^**−8**^	**0.71**	**1**
***Dusp5***	**Dual specificity phosphatase 5**	**3.05 × 10**^**−7**^	**1.03**	**1.71**
*Socs2*	Suppressor of cytokine signaling 2	3.80 × 10^−5^	1.16	1.47
*Trib2*	Tribbles homolog 2 (Drosophila)	8.93 × 10^−5^	1.1	1.51
*Plec*	Plectin	1.06 × 10^−4^	0.68	0.93
*Fam43a*	Family with sequence similarity 43, member A	2.35 × 10^−4^	1.1	1.49
*mt-Nd6*	Mitochondrially encoded NADH dehydrogenase 6	3.19 × 10^−4^	0.87	0.6
***Gadd45g***	**Growth arrest and DNA-damage-inducible 45 gamma**	**3.19 × 10**^**−4**^	**0.99**	**0.56**
*Neurl3*	Neuralized E3 ubiquitin protein ligase 3	4.49 × 10^−4^	1.16	1.36
***mt-Nd4l***	**Mitochondrially encoded NADH dehydrogenase 4L**	**1.21 × 10**^**−3**^	**0.69**	**1.25**
*Hsd17b7*	Hydroxysteroid (17-beta) dehydrogenase 7	1.35 × 10^−3^	1.03	1.7
***Chsy1***	**Chondroitin sulfate synthase 1**	**3.07 × 10**^**−3**^	**1.02**	**0.74**
*Klf2*	Kruppel-like factor 2 (lung)	3.10 × 10^−3^	1.52	1.43
*Chkb*	Choline kinase beta	4.37 × 10^−3^	1.19	1.37
***Arrdc4***	**Arrestin domain containing 4**	**6.09 × 10**^**−3**^	**0.94**	**1.64**
*Cdkn1a*	Cyclin-dependent kinase inhibitor 1A (P21)	7.17 × 10^−3^	1.47	1.55
*Bbs12*	Bardet-Biedl syndrome 12 (human)	1.56 × 10^−2^	0.92	0.67
*Itpripl2*	Inositol 1,4,5-triphosphate receptor interacting protein-like 2	1.84 × 10^−2^	0.84	0.77
*Gm6768*	Predicted gene 6768	1.86 × 10^−2^	0.92	0.83
*Tsc22d3*	TSC22 domain family, member 3	2.00 × 10^−2^	1.27	1.3
*Mat2a*	Methionine adenosyltransferase II, alpha	2.28 × 10^−2^	1.25	1.53
*Hist1h1d*	Histone cluster 1, H1d	2.30 × 10^−2^	0.98	1.63
*Sqstm1*	Sequestosome 1	2.57 × 10^−2^	1.16	1.37
*Gm10801*	Predicted gene 10801	2.57 × 10^−2^	1.39	1.02
***Zfp36l2***	**Zinc finger protein 36, C3H type-like 2**	**2.58 × 10**^**−2**^	**1.21**	**0.71**
***Ptger4***	**Prostaglandin E receptor 4 (subtype EP4)**	**2.77 × 10**^**−2**^	**1.08**	**0.75**
*Sox7*	SRY (sex determining region Y)-box 7	3.19 × 10^−2^	0.92	0.72
*Mad2l1bp*	MAD2L1 binding protein	3.65 × 10^−2^	1.37	1.2
*Hist3h2a*	Histone cluster 3, H2a	3.82 × 10^−2^	1.13	1.47
*2700097O09Rik*	RIKEN cDNA 2700097O09 gene	4.22 × 10^−2^	1.17	1.51

34 Candidate genes identified by RNA-seq are ordered by lowest false discovery rate (FDR).

^a^ Bolded genes had a treatment effect that differed between strains (C57BL/6J and LP/J).

^b^ FDR., False discovery rate. Significance was set at FDR≤0.05 for treatment effect (AgNP) and ≤0.1 for strain-dependent treatment effect (C57BL/6J vs. LP/J).

^c^ Fold change in high (C57BL/6J) and low responder (LP/J) strains following silver nanoparticle (AgNP) treatment compared to non-treated control.

### Validation of select candidate genes

**Fig 5:**
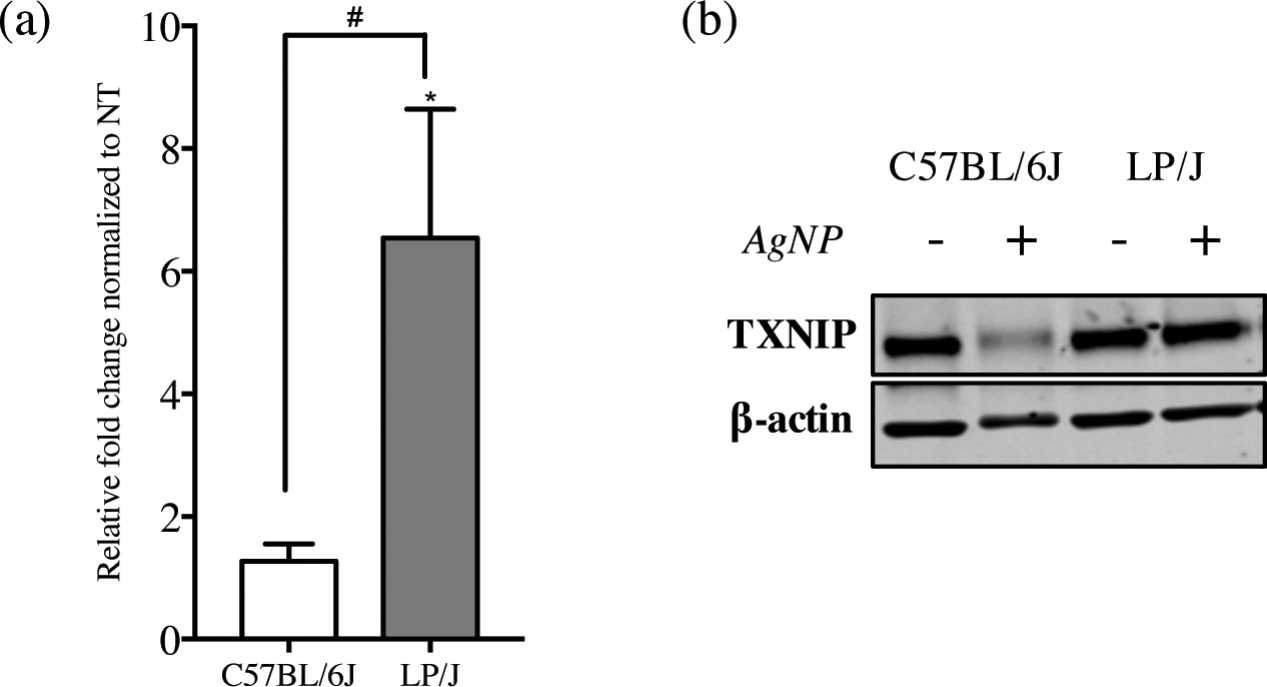
Expression of *Txnip* following AgNP treatment. Bone marrow-derived mast cells (BMMCs) were treated with AgNP for 1 h at 25 μg/ml and A) mRNA expression of *Txnip* was quantified by real-time quantitative polymerase chain reaction (qPCR). Values are expressed as fold change compared to non-treated cells (n = 3/group) normalized to *Gapdh*. B) Representative immunoblots of TXNIP expression in both high (C57BL/6J) and low (LP/J) responder strains following AgNP treatment (1 h at 25 μg/ml). Values are expressed as mean ± SEM of at least 3 independent experiments. * Indicates significant difference from controlled group (*p*≤ 0.05). # Indicates significant difference between strains (*p*≤ 0.05).

Expression of several AgNP-responsive genes were further validated to assess the reliability of our RNA-seq data set and identify potential gene biomarkers. Genes were chosen for a validation study based on their inclusion in the set of susceptible genes identified by association mapping in addition to the 34 AgNP-responsive genes (Fig 5). These genes include: *Fam43a*, *Trib2*, *Dusp5*, *Sqstm1*, and *Traf6*. qPCR analysis confirmed a significant difference (*p*<0.05) in effects of AgNP on expression between low and high responder strains for *Txnip* (Fig 5A). The remaining 6 genes demonstrated similar trends observed in the RNA-seq analysis, however the differences were not significant (S3 Fig). Consistent with the gene expression analysis, a significant decline in protein expression of TXNIP was observed following AgNP treatment in the high responder strain (C57BL/6J), where no observable effect was demonstrated in the low responder strain (Fig 5B).

**Fig 6:**
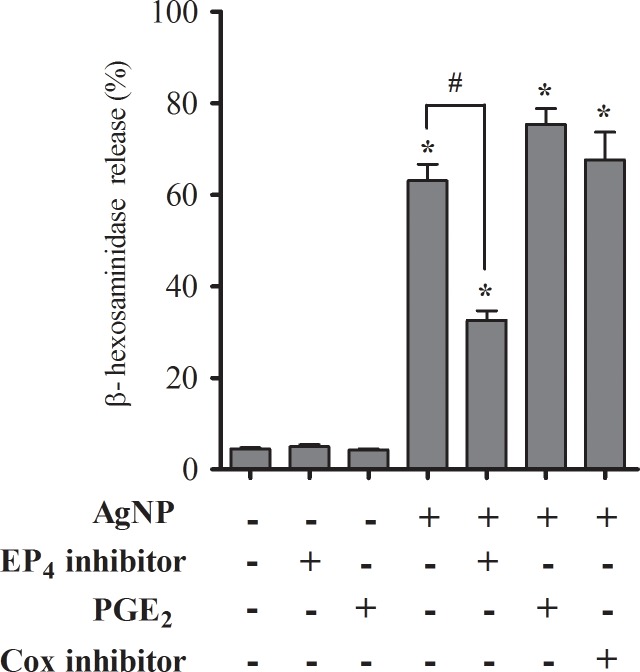
Prostaglandin receptor (EP_4_) expression and inhibition of AgNP-directed mast cell degranulation. Bone marrow-derived mast cell (BMMCs) isolated from C57BL/6J mice were evaluated for degranulation by measuring ß-hexosaminidase release following 1 h exposure of AgNP at 25 μg/ml ± pre-treatment with EP_4_ selective inhibitor (10 μM), prostaglandin (PGE_2_, 10 μM), or Cox-1 and -2 inhibitors (indomethacin, 5 μM). Values are expressed as mean ± SEM (n = 3). * Indicates significant difference compared to non-treated control and ^#^ indicates significant difference compared to AgNP treatment alone (*p* ≤ 0.05).

Functional studies were performed on the prostaglandin E receptor (EP_4_) which was identified in the RNA-seq analysis (*Ptger4*), and demonstrated a 25% decrease in gene expression following AgNP exposure in the low responder strain with no significant expression effect in the high responder strain (Table 2). To evaluate a plausible role for EP_4_ receptor function in mast cell degranulation, BMMCs isolated from C57BL/6J were assessed for degranulation following pre-treatment with EP_4_ antagonist and agonists. BMMCs pre-treated with an EP_4_ selective antagonist caused a significant reduction in AgNP-induced mast cell degranulation (Fig 6). In addition, no significant effect was observed following pre-treatment with PGE_2_, an EP_4_ ligand. Similar results were observed following pre-treatment with indomethacin, a nonselective inhibitor for COX-1 and -2, which prevents PGE_2_ synthesis (Fig 6). This suggests that AgNP may possibly be activating mast cells through an EP_4_-mediated mechanism, which is independent from PGE_2_ stimulation.

## Discussion

Using a systems biology approach, we aimed to identify the genetic factors and potential mechanisms of activation that are contributing to non-IgE-mediated mast cell degranulation by silver nanoparticles (AgNPs). In our study, utilizing mast cells from 23 strains of mice, we evaluated mast cell degranulation induced by AgNP exposure and compared the response to classical activation induced by crosslinking of the high affinity IgE receptor (e.g. FcεRI) by dinitrophenyl (DNP). Interestingly, we observed contrasting treatment-induced responses across strains indicating a distinct genetic component to AgNP directed mast cell degranulation. Following FcεRI stimulation, the highest responding strain was RIIIS/J, while the lower responder strain was C57BL/6J, a well-characterized strain commonly used in biomedical research. On the contrary, strain distribution was quite different following AgNP treatment, in which C57BL/6J was one of the highest responding strains. The dissimilar strain distribution patterns between the two treatments clearly indicates that AgNPs are activating mast cells via a novel mechanism compared to FcεRI crosslinking. Previous electron microscopy studies in our laboratory have demonstrated minimal uptake of AgNPs by mast cells, suggesting a potential mechanism mediated via surface interaction of the particle with a membrane receptor [11, 17]. Following bioinformatics analyses, putative risk loci associated with AgNP and DNP responses and represented susceptibility genes were reported. In addition, RNA sequencing (RNA-seq) of high and low responder strains identified a subset of AgNP-responsive genes that displayed a unique profile compared to DNP stimulation.

No previous genetic studies have identified mechanisms of mast cell degranulation by nanoparticle exposure; therefore, our interests were in the identification of novel genes of biological relevance to non-IgE mediated degranulation. An association mapping study of approximately 200,000 SNPs characterized across 22 strains (one strain was excluded) of mice identified 3 loci (located on chromosome 1, 2, and 18) associated with AgNP-induced mast cell degranulation compared to 1 non-overlapping locus (chromosome 9) associated with DNP stimulation (Bonferroni corrected *p*-value <0.05). The differences in associated loci between treatments, AgNP and DNP, further suggest that AgNP is likely mediated through a receptor-driven pathway distinct from IgE crosslinking leading to mast cell degranulation.

Several of the identified AgNP-associated loci (Chromosome 2 and 18) in the current study have previously been implicated in other association mapping studies concomitant with circulating toxic metals [50] and immune-mediated diseases [51–54]. Interestingly, the AgNP-associated loci identified *Trak2* and *Traf6* on chromosomes 1 and 2, previously reported together as genetic variants associated with several common inflammatory diseases including atopic dermatitis, psoriasis, and rheumatoid arthritis [47–49]. *Traf6* is an essential signal transducer downstream from tumor necrosis factor (TNF) receptor and toll-like receptor (TLR) superfamily members and has been shown to contribute to mast cell activation [55, 56]. Interestingly, *Traf6*-deficient mast cells demonstrate normal IgE-mediated degranulation, raising the possibility that this protein may regulate alternative pathways involved in non-IgE-mediated degranulation. For example, Madera-Salcedo et. al., demonstated a role for a Traf6/ß-arrestin complex in suppressing mast cell activation by modulating opioid receptors, a class of G protein-coupled receptors (GPCRs) that recruit ß-arrestins and mediate signaling pathways leading to inhibition of Ca^2+^ channels [57, 58]. Taken together, we postulate that GPCR activation may be contributing to AgNP-mediated mast cell degranulation.

Additionally, the loci on chromosome 1 contains a cohort of biologically interesting genes (*Cflar*, *Clk1*, *Ndfufb3*, *Nif3l1*, *Bzw1*, *Ppil3*, *Casp8*, *Fam126b*) recently reported in another association mapping study to be predictive markers in cellular toxicity to triptolide, an anti-inflammatory and immunosuppressive compound commonly used in traditional Chinese medicine to treat several immune complex diseases such as rheumatoid arthritis and systemic lupus erythematous [59, 60]. Studies have shown that triptolide can alter multiple signaling pathways leading to the down-regulation of c-Kit and inhibition of nuclear factor-kappa B (NF-κB) and phosphoinositide 3-kinase (PI3K) signaling, key cell signaling molecules in mast cell activation [61, 62]. These findings may be important, as we have recently reported that PI3K phosphorylation is required to elicit mast cell degranulation by AgNPs [17], however no functional data exists for many of the identified genes in evaluating their respective roles in mast cell degranulation and therefore warrants further study.

Using RNA-seq for the first time in murine mast cells exposed to DNP or AgNP, we have uncovered genetic targets involved in non-IgE mediated mast cell responses compared to the traditional activation by FcεRI cross-linking. Transcriptomic analysis identified a combined 3890 DE genes following exposure to either DNP or AgNP. Interestingly, many of the genes were DE following DNP treatment (3805), indicating the abundant gene expression changes occurring following stimulation with antigen crosslinking of the IgE receptor. Although interesting, we chose to focus our current study on the DE genes following AgNP treatment in the aim of better understanding mechanisms of activation by non-IgE triggers. In so forth, the 34 DE genes in response to AgNP were the major focus of the discussion, with special attention on the 11 genes displaying DE patterns that differed between the high and low responder strains.

Statistical enrichment analysis of the 34 AgNP-responsive genes demonstrated that many of the genes were involved in stress response and signal transduction (Fig 4C). Central to these responses are *Txnip*, *Arrdc3* and *Arrdc4*, all three are members of the arrestin superfamily which have been shown to both negatively and positively regulate GPCR signaling [63, 64]. In addition to their previously mentioned interaction with Traf6, they are also involved in complement receptor C3aR desensitization and NF-κB activation in mast cell degranulation [65]. A member of the α-arrestin family, thioredoxin interacting protein (*Txnip*), (also called vitamin D3 up-regulated protein (VDUP1) and thioredoxin-binding protein-2 (TB-2)) may play a protective role in AgNP-induced mast cell degranulation. We observed a 300% increase in expression in the low responder strain (LP/J) in comparison to minimal expression changes in the high responder strain (C57BL/6J) in response to AgNP. *Txnip* encodes a multifunctional protein that has emerged as a key component of cellular redox balance by inhibiting the reducing activity of thioredoxin (TRX) [66]. It has been established that oxidative stress can contribute to IgE-mediated mast cell degranulation, as observed by a significant suppression of histamine release by FcεRI crosslinking in a TRX-transgenic mouse model compared to wild-type [67]. In addition to redox-dependent functions, Txnip also functions as an arrestin protein modulating expression and function of key receptor(s) and downstream signal transduction pathways [68].

We have recently reported that AgNPs are not readily taken up in mast cells, rather electron microscopy imaging suggests that initiation of mast cell degranulation appears to be membrane or receptor-mediated [17]. Therefore, we hypothesize that Txnip may be regulating a key receptor in the alternative, non-IgE mediated mast cell degranulation pathway. In the transcriptomic study, we identified the prostaglandin E receptor 4 (EP_4_) as an AgNP-responsive gene (*Ptger4*). This gene warrants further analysis through knockout or knockdown studies as it was the only receptor identified in the transcriptomic analysis. EP receptor subtypes 1–4 respond to prostaglandin (PGE_2_) and activate various signaling pathways [69]. An arachidonic acid metabolite, PGE_2_ functions as both a pro- and anti-inflammatory lipid mediator that balances T_H_1/T_H_2 responses via stimulation of different EP receptors. In relation to mast cell degranulation, different receptor subtypes vary the biological response. PGE_2_-mediated activation of EP_3_ and EP_4_ receptors can induce mast cell degranulation by increasing intracellular Ca^2+^, while EP_2_ stimulation has the opposite effect as shown by inhibition of IgE-mediated histamine release in human lung mast cells [70–72]. Additionally, Nguyen et. al. observed a genetic effect in PGE_2_-induced inflammation by mast cells [73]. Currently, we demonstrated that BMMCs pre-treated with a selective EP_4_ antagonist partially, but significantly inhibited AgNP-induced mast cell degranulation in the high responder strain (C57BL/6J; Fig 6). Additionally, there was no significant effect following pre-treatment with PGE_2_ or indomethacin. This suggests that AgNPs may be indirectly mediating a response via EP_4_ activation however the exact mechanism is not known and should be further investigated with additional functional studies using knockout or knockdown models. We have previously reported a role for scavenger receptor B1 (SR-B1) in mast cell degranulation by AgNPs, however this receptor was not identified in our transcriptomics analysis. Our statistical analyses were utilized to identify a subset of genes with expression patterns that significantly differed between strains, however a shortcoming in the study may have missed genes that were significantly expressed in both strains. Also, the lack of identification could be due to the short time frame of the study (1 hr) [10].

Taken together, we conclude that AgNP-induced mast cell degranulation is a complex process that is likely dependent on many factors, potentially mediated through the regulation of GPCRs upstream of key signaling cascades. Most research efforts are focused on mast cell signaling through the high affinity IgE receptor (FcεRI), although an alternative pathway may contribute in relation to AgNP exposure which may involve GPCR-mediated activation of PI3K and MAPK signaling cascades [62]. Still to date, no studies have linked GPCR activation to nanoparticle-induced allergic inflammation. Therefore, it may be of interest to target GPCR-regulators, such as the α/β-ARR family members in future studies. Txnip may be a plausible therapeutic target because of its dual role in redox-dependent and redox-independent regulation of GPCR-mediated signaling pathways. For example, treatment with an anti-asthmatic drug, eugenol, led to the up-regulation of Txnip and down-regulation of NF-κB, ameliorating the progression of asthma in an OVA-induced mouse model [74]. In addition, we observed a novel connection between EP_4_ activation and mast cell degranulation by a non-IgE trigger. However, future functional studies need to be performed to validate the genes identified, which is beyond the scope of this study. By our knowledge, this is the first study performed combining bioinformatics analyses to study the association of genetic variants by association mapping and transcriptional changes by RNA-sequencing to determine a mechanism of mast cell activation by a non-IgE trigger. Association mapping has become a powerful tool in reproducibly identifying genetic regions associated with common disease traits due to recent advances in genotyping and publicly available SNP databases [75]. RNA-sequencing is gaining traction as means to characterize genomic expression and has been utilized in numerous studies to acquire knowledge about mechanisms of complex diseases [76]. These complementary analyses offer additional insights into novel disease pathology that one test alone could not infer. This study has provided novel gene targets and proposed mechanisms to better understand non-IgE mediated mast cell degranulation in addition to addressing the emerging concern regarding nanoparticle exposures and human health and safety.

## Supporting information

S1 Fig Surface expression of mature bone marrow derived mast cells (BMMCs). FcεRI (white bar) and c-Kit (grey bar) were analyzed on the surface of BMMCs at week 5 via flow cytometry. Values are expressed as mean ± SEM (n = 2-3/group).
(TIF)

S2 Fig Dose-dependent increase in human mast cell degranulation following AgNP exposure. Human mast cells (LUVA; Kerafast) were evaluated for degranulation by measuring ß-hexosaminidase release following 1 h exposure of AgNP at 25 and 50 μg/ml. Values are expressed as mean ± SEM of at least 3 independent experiments. * Indicates significant difference from controlled group (*p*≤ 0.05).
(TIFF)

S3 Fig Expression of selected AgNP-responsive genes. Bone marrow-derived mast cells (BMMCs) were stimulated with AgNP for 1 h at 25 μg/ml and the mRNA expression of select genes was quantified by real-time quantitative polymerase chain reaction (qPCR). Values are expressed as fold change compared to non-treated cells (n = 3/group) normalized to *Gapdh*. Values are expressed as mean ± SEM of at least 3 independent experiments.
(TIF)

S1 Table RNA-seq data for genes differentially expressed following AgNP and DNP treatment. Ensembl gene IDs and MGI gene symbols are reported. *Indicates *p*-value of treatment. **Indicates *p*-value of strain-dependent treatment effect. Chr., chromosome. Start, chromosomal start.
(XLSX)
