# Supplementary figures and images for: Genomic and transcriptomic comparison of allergen and silver nanoparticle-induced mast cell degranulation reveals novel non-immunoglobulin E mediated mechanisms
Source: PLoS One. 2018 Mar 22;13(3):e0193499. doi: 10.1371/journal.pone.0193499 (PMC5863960; doi:10.1371/journal.pone.0193499)

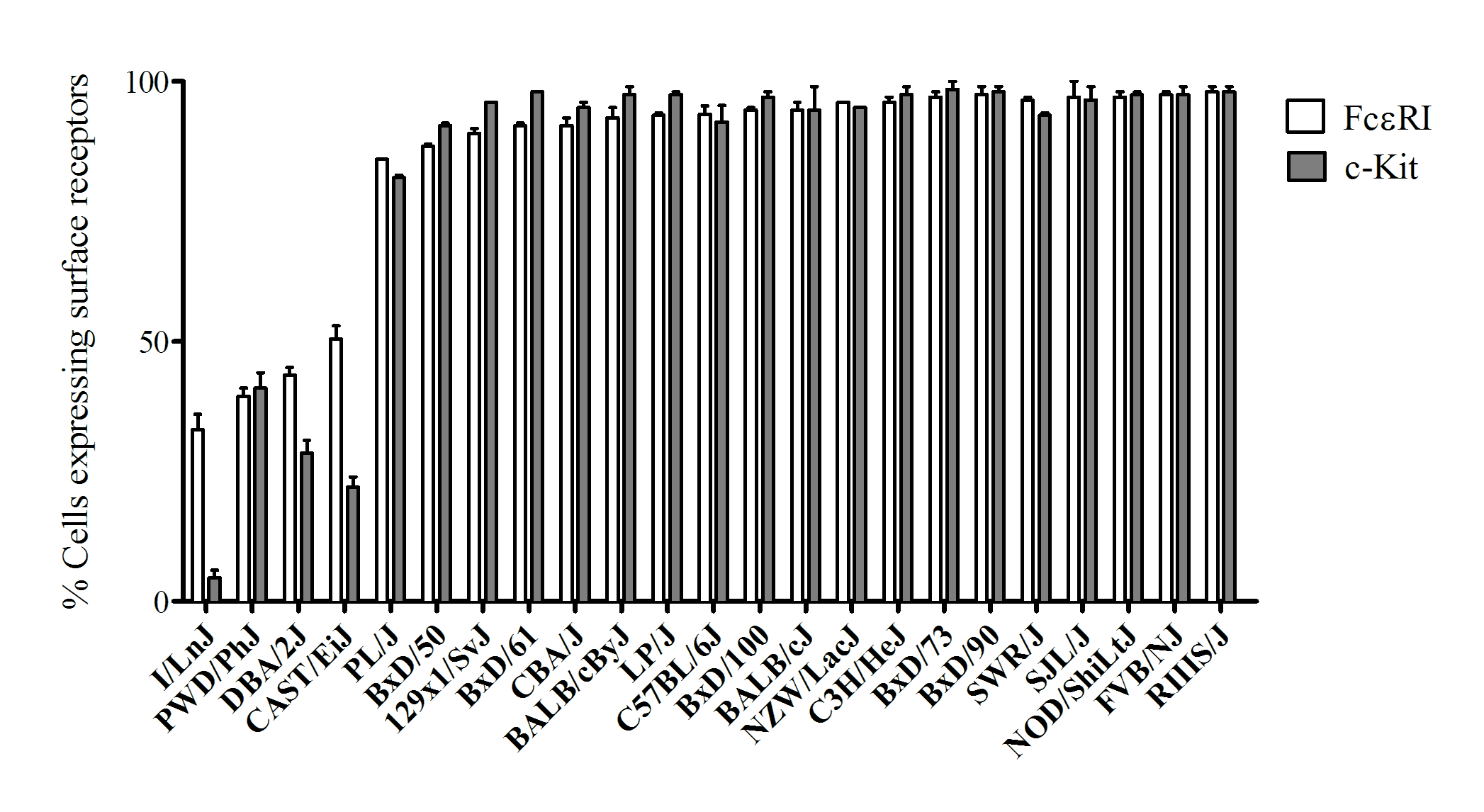

Supplement: S1 Fig — FcεRI (white bar) and c-Kit (grey bar) were analyzed on the surface of BMMCs at week 5 via flow cytometry. Values are expressed as mean ± SEM (n = 2-3/group). (TIF) [file pone.0193499.s001.tif]

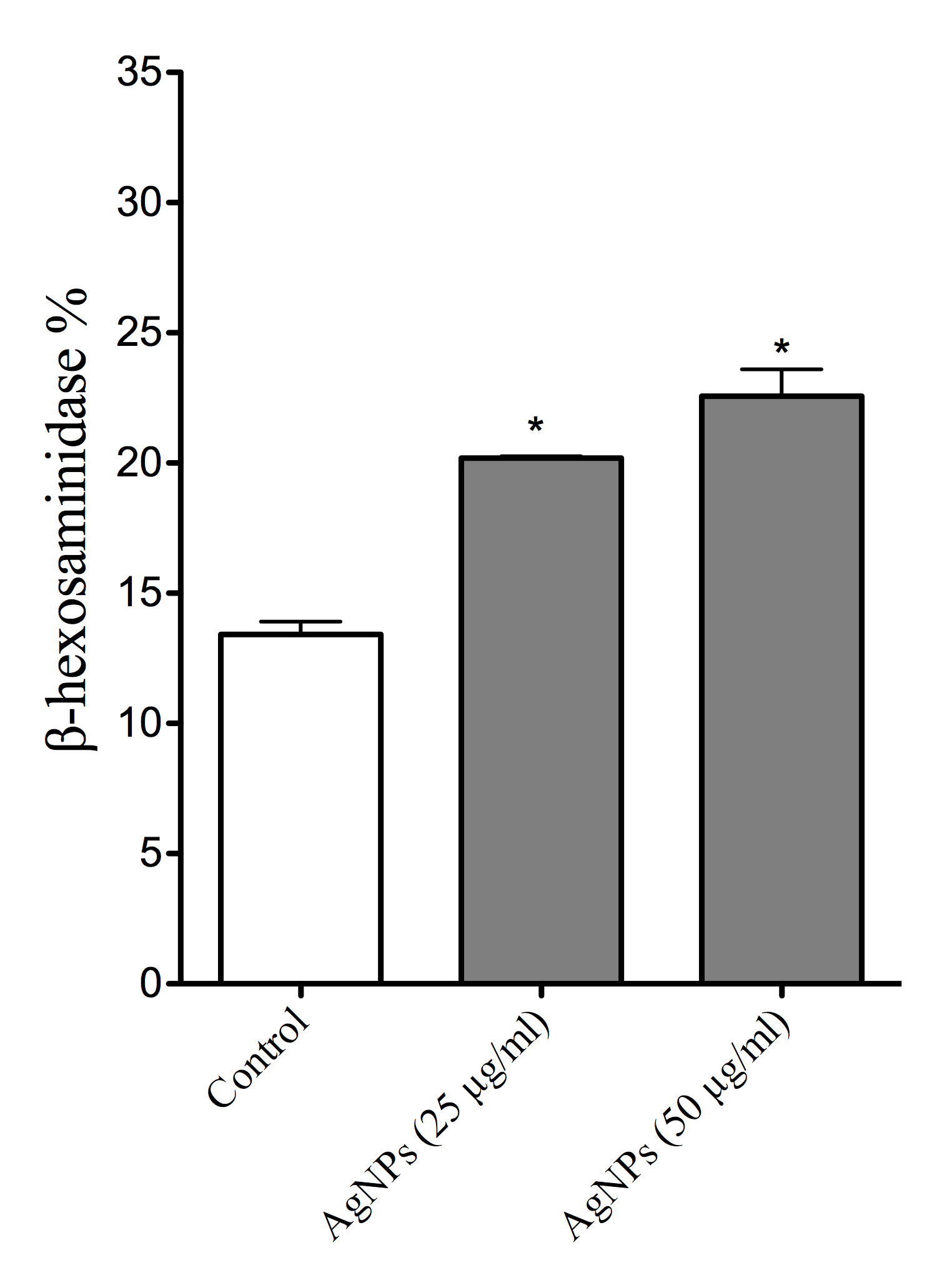

Supplement: S2 Fig — Human mast cells (LUVA; Kerafast) were evaluated for degranulation by measuring ß-hexosaminidase release following 1 h exposure of AgNP at 25 and 50 μg/ml. Values are expressed as mean ± SEM of at least 3 independent experiments. * Indicates significant difference from controlled group (p≤ 0.05). (TIFF) [file pone.0193499.s002.tiff]

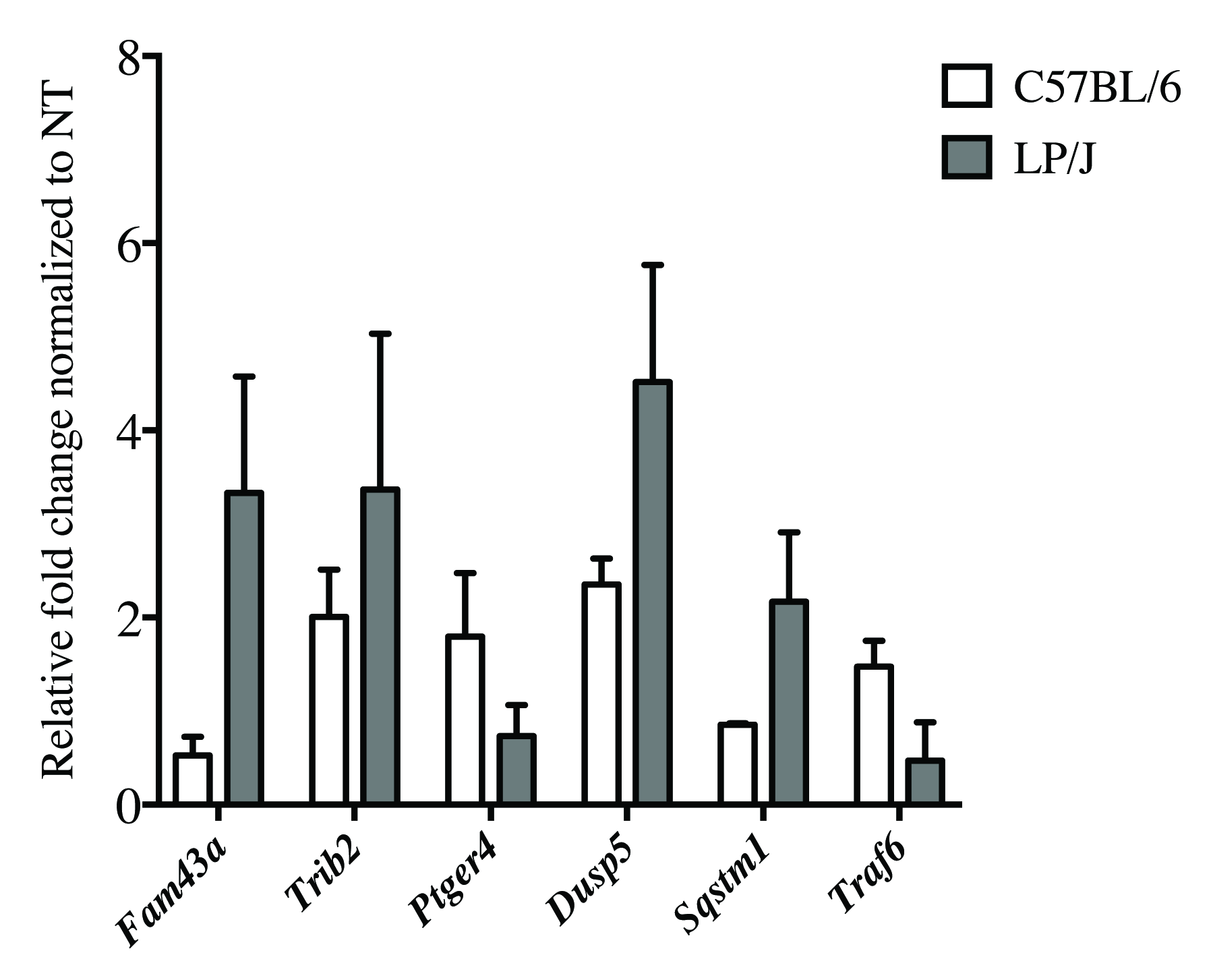

Supplement: S3 Fig — Bone marrow-derived mast cells (BMMCs) were stimulated with AgNP for 1 h at 25 μg/ml and the mRNA expression of select genes was quantified by real-time quantitative polymerase chain reaction (qPCR). Values are expressed as fold change compared to non-treated cells (n = 3/group) normalized to Gapdh. Values are expressed as mean ± SEM of at least 3 independent experiments. (TIF) [file pone.0193499.s003.tif]
